# Trainees’ perceptions and expectations of formal academic mentoring during the COVID-19 pandemic in Indonesian cardiology residency programs

**DOI:** 10.3352/jeehp.2021.18.19

**Published:** 2021-08-09

**Authors:** Sunu Budhi Raharjo, Rita Mustika, Aida Lydia, Mefri Yanni, Heru Sulastomo, Rahma Tsania Zhuhra, Celly Anantaria Atmadikoesoemah

**Affiliations:** 1 Department of Cardiology and Vascular Medicine, Faculty of Medicine, Universitas Indonesia, Jakarta, Indonesia; 2 Medical Education Collaboration Cluster, Indonesian Medical Education and Research Institute, Universitas Indonesia, Jakarta, Indonesia; 3 Department of Medical Education, Faculty of Medicine, Universitas Indonesia, Jakarta, Indonesia; 4 Kidney and Hypertension Division, Department of Internal Medicine, Universitas Indonesia, Jakarta, Indonesia; 5 Department of Cardiology and Vascular Medicine, Faculty of Medicine, Andalas University, Padang, Indonesia; 6 Department of Cardiology and Vascular Medicine, Faculty of Medicine, Sebelas Maret University, Surakarta, Indonesia; Hallym University, Korea

**Keywords:** Communication, COVID-19, Indonesia, Internship and residency, Mentoring

## Abstract

**Purpose:**

During medical residency programs, physicians develop their professional identities as specialists and encounter high expectations in terms of achieving competencies. The responsibilities of medical trainees include caring for patients, balancing work with personal life, and weathering stress, depression, and burnout. Formal academic mentoring programs strive to ease these burdens. The coronavirus disease 2019 (COVID-19) pandemic has altered the trainee–academic mentor relationship, and solutions are needed to address these challenges. The present study aimed to evaluate the formal academic mentoring process through trainees’ perceptions and expectations of formal mentoring programs during COVID-19 in Indonesian cardiology residency programs.

**Methods:**

This cross-sectional study used a self-administered online questionnaire to capture trainees’ perceptions and expectations regarding academic mentoring programs in 3 cardiology residency programs in Indonesia from October to November 2020. The questionnaire was developed before data collection. Perceptions of the existing mentoring programs were compared with expectations.

**Results:**

Responses were gathered from 169 out of 174 residents (response rate, 97.3%). Most trainees reported having direct contact with COVID-19 patients (88.82%). They stated that changes had taken place in the mode and frequency of communication with their academic advisors during the pandemic. Significant differences were found between trainees’ perceptions of the existing mentoring programs and their expectations for academic mentoring programs (P<0.001).

**Conclusion:**

Despite the challenges of interacting with their academic mentors, trainees still perceived academic mentors as a vital resource. Study programs need to consider trainees’ expectations when designing academic mentoring programs.

## Introduction

### Background/rationale

Formal academic mentoring has been employed in medical residency programs, with favorable impacts [1-3]. Proper academic mentoring creates a humanistic learning climate that supports the formation of trainees’ professional identity [4]. Trainees have reported that these programs are helpful in both their professional and personal lives [1,3]. In medical residency programs, trainees are pushed to learn primarily through the socialization process in their learning environment. The academic mentor shares wisdom and helps them solve the problems they face academically and personally [1-4]. In a study conducted in a Korean setting, Chun et al. [5] found that a formal mentoring relationship was mutually beneficial for both mentors and mentees, who experienced improvements in their effective well-being and organizational commitment. Moreover, formal mentoring also increased transformational leadership behavior in mentors [5]. The structure of formal academic mentoring varies among study programs and institutions. Generally, a residency program assigns an academic mentor to a trainee after admission and requires them to meet frequently throughout residency. Cardiology residency programs in Indonesia—like other residency programs—are required to provide formal academic mentoring. From the beginning of the residency, every trainee has an academic mentor. They are instructed to meet at least once a semester, and the academic mentor is responsible for monitoring and helping his or her mentee throughout the residency period.

The coronavirus disease 2019 (COVID-19) pandemic caused substantial changes in most medical training processes, including residency programs. In the context of cardiology, medical staff and trainees were moved to the front line to treat cardiologic conditions in patients with COVID-19. Patients with cardiologic problems often limited their hospital visits to minimize the risk of COVID-19 infection, leading to fewer cases for trainees. COVID-19 has resulted in increased physical distancing, limited practice opportunities, difficulties in fulfilling competencies in the given time, and an increased risk of isolation, depression, and anxiety. However, opportunities have also arisen in these challenging circumstances. Cardiology trainees have had opportunities to manage cardiac complications related to COVID-19, learn new skills, work with interdisciplinary teams, adapt to telemedicine, and create opportunities for research due to the lack of patients with specific cardiology-related conditions [6].

The changes wrought by the pandemic have also altered the relationship between trainees and academic mentors. Physical distancing, personal protective equipment requirements, and limitations in practicing medicine have made direct communication burdensome. The mentoring process thus needs to adapt to these changes.

### Objectives

This study aimed to evaluate the formal academic mentoring process in cardiology residency programs in Indonesia through trainees’ perceptions of existing formal mentoring programs during the COVID-19 pandemic and their expectations of such programs.

## Methods

### Ethics statement

This study was granted ethical approval from the Research Ethics Committee of the National Vascular Centre ‘Harapan Kita’ Jakarta, Indonesia (no., LB.02.01/VII/439/KEP. 044/2020). Informed consent was obtained in the first section of the online questionnaire. All participants were asked to consent to the use of data from this study, and their anonymity was preserved.

### Study design

This cross-sectional study employed an online questionnaire. The description was based on the STROBE (Strengthening the Reporting of Observational Studies in Epidemiology) statement [7].

### Setting

We included cardiology residency programs at 3 institutions in 3 cities in Indonesia: Universitas Indonesia (UI) in Jakarta, Andalas University (AU) in Padang, and Sebelas Maret University (SMU) in Surakarta. The National Cardiology College provides the curriculum and organizational guidance for the formal mentoring programs at all cardiology residency programs in Indonesia. We distributed the online questionnaire to all trainees from October to November 2020. In November 2020, we completed the data collection and initiated the data analysis.

### Participants: inclusion and exclusion criteria

All active trainees from 3 institutions who provided consent and completed the questionnaire were included in this study. We excluded incomplete questionnaires.

### Variables

The variables assessed in this study were trainees’ perceptions of their existing formal academic mentoring programs and their expectations for such programs. We evaluated the significance of differences between the 2 sets of variables (perceptions versus expectations) using the Mann-Whitney test.

### Data sources/measurement: validity and reliability of the questionnaire

Before data collection, we developed the questionnaire used to assess respondents’ perceptions of their formal mentoring programs, following the established steps of questionnaire development for medical education research [8]. Initially, we collected qualitative data through focus group discussion (FGDs) with 6 stakeholders from 3 residency programs and searched the literature. Data from the FGDs and literature review were used to develop the questionnaire items. The first draft of the questionnaire went through expert review, with 5 experts involved in the process. A content validity index of 1 was obtained, indicating that the questionnaire was valid. We distributed the questionnaire and calculated the internal consistency coefficient to be 0.97, showing that the questionnaire was reliable. The final questionnaire consisted of 18 items with a 7-point Likert scale from 1 (strongly disagree) to 7 (strongly agree) (Supplement 1).

### Study size

The target population of this study was cardiology trainees in Indonesian programs, and the accessible population constituted trainees from UI, AU, and SMU. We calculated the sample size using Statistics Kingdom (Melbourne, Australia), an online sample size calculator, based on 90% power, a medium effect size, and a 5% significance level for 18 predictors; the required sample size was 120.

### Bias

Since our study obtained a 97.3% response rate, there was a low risk of selection and performance bias. Moreover, we utilized a specially developed and validated instrument for data collection to eliminate measurement bias.

### Statistical methods

Data analysis was performed using IBM SPSS ver. 20.0 (IBM Corp., Armonk, NY, USA). Initially, demographic data were descriptively analyzed. We checked the prevalence of the items descriptively. Since the data were not normally distributed, we analyzed the differences between variables using the Mann-Whitney test.

## Results

### Participants and descriptive data

In total, 169 trainees consented to participate and answered the questionnaire: 90 from UI, 45 from AU, and 34 from SMU. The response rate was 97.3%. Males made up 59% of the respondents, representing the actual gender distribution in these 3 programs. As shown in Table 1, the respondents were roughly evenly distributed across the 3 years of the residency training.

### Main results

#### Trainees’ confrontation with the COVID-19 pandemic

Most trainees (88.82%) reported directly treating COVID-19 patients. Nonetheless, more than half of the trainees (51.47%) reported receiving sufficient resting time. Access to academic mentors was largely assessed as adequate (61.18%). Before the pandemic, most trainees (65.28%) met with their academic mentors once a week, although this proportion decreased to 45.4% during the pandemic. Moreover, before the pandemic, only 6.83% of trainees did not contact their academic mentors, but during the pandemic, this proportion increased to 26.11%. Before the pandemic, mentor-mentee interactions mainly occurred through face-to-face meetings (87.54%), but during the pandemic, most trainees’ interactions with their academic mentors shifted to WhatsApp, e-mail, and other social media platforms (83.98%), and trainees indicated that interacting one-on-one was preferable (68.55%). The training process, including rotation schedules, examinations, and daily tasks, dominated the discussions during mentoring sessions (56.68%), along with research/thesis preparation (46.88%). Trainees rarely reported discussing family problems with their academic mentors (0.30%) (Dataset 1).

#### Trainees’ perceptions and expectations of formal academic mentoring programs

Statistically significant differences were found between participants’ perceptions of the existing formal mentoring programs during the pandemic and their expectations for such programs in 16 of the 18 questionnaire items. Consistent scores between perceptions and expectations were only found for the items stating that the study program chose their mentor and that this appointment takes place after the student is admitted to the residency program. The highest score for perceptions was found for the item “the academic supervisor helps the trainee to choose a research topic” (5.68). In contrast, regarding expectations, the highest score was found for the item “the academic supervisor is easy to meet and contact when needed” (6.02). The questionnaire items are presented in Table 2.

## Discussion

### Key results

This study aimed to evaluate formal mentoring in cardiology residency programs. Our study found that trainees had high expectations of the formal mentoring programs, and some adjustments are needed. The COVID-19 pandemic changed trainees' daily activities and their interactions with mentors. Efforts to treat and prevent COVID-19 have altered human interactions in general. Moreover, social distancing, mask-wearing, and other health protocols have limited direct face-to-face contact. This study’s 3 key results include the matching of students with academic mentors, expectations regarding academic mentors’ performance, and support for professional development.

### Interpretation

Since the World Health Organization declared COVID-19 a pandemic in early 2020, there have been tremendous disruptions in medical education, including residency programs. Our study shows that formal mentoring in cardiology residency programs was particularly important before the pandemic as a way to support trainees in academic and clinical training; specifically, the mentor helped the trainee to find research topics and conducted routine evaluations of the trainee’s academic performance. During the pandemic, the mentoring program was expected to provide more comprehensive support. Evidence has also shown that for mentoring programs to be beneficial, trainees need to be assertive, as they initiate most meetings. Mentors themselves play an essential role in the success of formal academic mentoring programs, and mentors should have the ability to create a psychologically safe environment.

### Matching students with academic mentors

We found that the administrator of the cardiology residency program matched each trainee with an academic mentor at the beginning of the residency program. This practice is deemed appropriate since there was no substantial difference between trainees’ perceptions and expectations. However, trainees expected to select their own academic mentors although they agreed that the administration chose their academic mentors. Moreover, trainees reported that they would like to have the opportunity to request a different mentor if both parties agree. Clinical teachers who serve as academic mentors have multiple tasks and must find the time to meet with their mentees. Programs need to provide assistance by requiring mentors to meet with and monitor their mentees every semester.

### Academic mentors’ assistance

Trainees in residency programs come from various backgrounds, ranging from brand-new graduate medical practitioners to experienced physicians who have been practicing medicine for some time. They must adapt to the new environment, and the results of this study show that academic mentors are expected to assess trainees’ adaptability and be ready to assist in their adaptation. They are also expected to help trainees identify their strengths and weaknesses to develop competencies and overcome shortcomings. During residency, the mentoring process aims to provide a routine evaluation of academic performance, assess trainees’ potential and interests, and identify any problems that may occur.

From time to time, trainees need academic mentors to help them through the residency process. Academic mentors are expected to provide access to counseling and career planning. As the COVID-19 pandemic has created uncertainty in residency programs, mental support from mentors is even more necessary. In cardiology residency programs in Indonesia, trainees are required to complete a research thesis, and academic mentors are expected to guide the mentee through this process. An academic mentor is expected to help choose the research topic, monitor the research progress, and provide feedback.

### Professional development of residents

The residency program is when trainees’ professional identity develops from a general practitioner to a specialist. Academic mentors are expected to facilitate this development of proficiency and competency; they are also expected to help trainees expand their network and career. An academic mentor provides constructive feedback and acts as a role model, potentially inspiring the resident to aspire to a high level of professionalism.

### Comparison with previous studies

Cardiology residency programs have been strongly affected by the COVID-19 pandemic, leading to many changes [4,9]. Similar to our study, several studies have highlighted the importance of formal mentoring in residency programs [1,3,4]. The COVID-19 pandemic has led to several changes in cardiology residency programs like ours; trainees have been directly exposed to COVID-19 patients, and interactions between trainees and clinical teachers have diminished. Mentoring programs in the United States and other countries also use more online approaches than face-to-face meetings [2,10,11]. However, unlike our study, several mentoring programs in the United States use group models, whereas the trainees in our study preferred a personal mentoring model. Even though a group model provides peer support within the group, a personal mentoring model is more manageable in the pandemic situation. Similar to the programs in this study, several other mentoring programs match mentors and mentees at the beginning of residency [1,2,4,6]. In our study, trainees met their mentors at least once per semester (twice a year), but in another report in the United States, the mentoring program for residents has meetings scheduled 4 times a year [11]. A study by Andrades et al. [1] in 2013 at the Aga Khan Medical School highlighted the importance of an excellent relationship between the mentor and mentee for the effectiveness of formal mentoring programs. Similar to our findings, that study reported a mentor’s essential attributes to be accessibility, active listening, support for emotional and psychological needs, and trust in the relationship [1].

### Limitations

In this study, we evaluated formal academic mentoring programs by comparing trainees’ perceptions of existing programs with their expectations. Adding data from other resources and using longitudinal research or qualitative/phenomenological research methods would provide valuable information to evaluate the programs more comprehensively.

### Generalizability

In response to COVID-19, trainees in most medical residency programs were moved to front-line positions to fight the pandemic, which has led to an increased prevalence of mental health challenges. Mentoring programs are considered to be a critical support system for trainees. Cardiology residency programs are among those that have been altered by the pandemic. This study conducted a total sampling of 3 different cardiology programs in different cities in Indonesia and employed a valid instrument, making the results generalizable to cardiology residency programs nationwide in Indonesia. Expanding the target population in future research would enhance generalizability to other residency programs.

### Suggestions

The results of this study provide essential feedback for improving formal mentoring programs. Further research on how to restructure these programs using the results of this study would be beneficial.

### Conclusion

Academic mentoring in residency programs is changing due to the COVID-19 pandemic. The majority of trainees have been directly exposed to COVID-19 cases while working on the front lines. The pandemic has altered the clinical learning environment and communication between residents and mentors, leading to changes in the mentoring process and mentor roles. The effectiveness of formal academic mentoring programs could be improved based on residents’ expectations.

## Figures and Tables

**Table 1.** Characteristics of the respondents (N=169)

Characteristic	Cardiology UI (N=90)	Cardiology AU (N=45)	Cardiology SMU (N=34)
Age (yr)	30.39±2.15	30.71±2.76	31.50±2.48
Gender			
Female	37 (41.1)	21 (46.7)	12 (35.3)
Male	53 (58.9)	24 (53.3)	22 (64.7)
Stage of education			
Year 1	33 (36.7)	22 (48.9)	12 (35.5)
Year 2	31 (34.4)	14 (31.1)	11 (32.4)
Year 3	26 (28.9)	9 (20.0)	11 (32.4)

Values are presented as mean±standard deviation or number (%). UI, Universitas Indonesia (in Jakarta); AU, Andalas University (in Padang); SMU, Sebelas Maret University (in Surakarta).

**Table 2.** Trainees’ perceptions and expectations of the formal academic mentoring programs

Items	Perceptions	Expectations	P-value
1. The academic supervisor is selected by the study program	5.90±1.38	5.85±1.37	0.619
2. The study program appoints the academic supervisor soon after the trainee’s admission	5.92±1.36	5.88±1.33	0.318
3. A trainee submits the expected name of his or her academic supervisor to the study program	3.81±2.02	5.04±1.80	<0.001
4. The academic supervisor assesses the adaptability of the new trainees and helps them in the adaptation process during training	5.01±1.46	5.77±1.23	<0.001
5. Changing to a different academic supervisor is possible if both parties feel that the relationship between the two is not going well	4.77±1.60	5.61±1.37	<0.001
6. The study program requires the academic supervisor to meet with the trainee at least once a semester	5.32±1.54	5.80±1.32	<0.001
7. The academic supervisor conducts routine evaluations of the trainee’s academic performance	5.11±1.51	5.80±1.26	<0.001
8. The academic supervisor helps the trainee identify his or her strengths and weaknesses	5.07±1.51	5.80±1.33	<0.001
9. The academic supervisor provides information on access to resources and support in career planning	5.19±1.52	5.87±1.30	<0.001
10. The academic supervisor provides information on access to resources and counseling services	5.21±1.48	5.87±1.35	<0.001
11. The academic supervisor knows the trainee’s life situation and its impact on education and performance	5.16±1.48	5.78±1.31	<0.001
12. The academic supervisor understands the trainee’s potential and interest	5.08±1.44	5.81±1.27	<0.001
13. The academic supervisor is easy to meet and contact when needed	5.71±1.23	6.02±1.21	<0.001
14. The academic supervisor helps the trainee to become professional and competent	5.59±1.29	5.92±1.23	<0.001
15. The academic supervisor helps the trainee to expand their network and career experience	5.40±1.36	5.88±1.26	<0.001
16. The academic supervisor helps the trainee to choose a research topic	5.68±1.35	5.80±1.36	<0.001
17. Having an academic supervisor helps the trainee through the educational process	5.19±1.48	5.89±1.26	<0.001
18. During the COVID-19 pandemic, the academic supervisor provided support and identified problems such as stress and burnout in trainees	5.00±1.53	5.80±1.33	<0.001

Values are presented as mean±standard deviation. COVID-19, coronavirus disease 2019.

## Footnotes

**Data availability**
Data files are available from Harvard Dataverse: https://doi.org/10.7910/DVN/0CNPTA Dataset 1. Raw response data of survey from residents for the perceptions of their existing and expected formal mentoring programs during COVID-19 in Indonesian cardiology residency programs.

## Supplementary materials

Data files are available from Harvard Dataverse: https://doi.org/10.7910/DVN/0CNPTA

Supplement 1. Full questionnaire distributed to the cardiology residents.

Supplement 2. Audio recording of the abstract.
