# Supplementary material for: Trainees’ perceptions and expectations of formal academic mentoring during the COVID-19 pandemic in Indonesian cardiology residency programs
Source: J Educ Eval Health Prof. 2021 Aug 9;18:19. doi: 10.3352/jeehp.2021.18.19 (PMC8616722; doi:10.3352/jeehp.2021.18.19)
Supplement: Supplementary file 2 — Supplement 1. Full questionnaire distributed to the cardiology residents. [file jeehp-18-19-suppl1.docx]

Tables

**Table 1. Characteristics of Respondents (N=169)**

| **Characteristics** | **Cardiology UI**  **(N = 90)** | | **Cardiology AU**  **(N = 45)** | | **Cardiology SMU**  **(N = 34)** | |
| --- | --- | --- | --- | --- | --- | --- |
| **Age (year)** | 30.39 (2.15) | | 30.71 (2.76) | | 31.50 (2.48) | |
| **Gender (%)** |  |  |  | |  | |
| **Female** | 37 | (41.1) | 21 | (46.7) | 12 | (35.3) |
| **Male** | 53 | (58.9) | 24 | (53.3) | 22 | (64.7) |
| **Stage of education (%)** |  |  |  |  |  |  |
| **Stage 1** | 33 | (36.7) | 22 | (48.9) | 12 | (35.5) |
| **Stage 2** | 31 | (34.4) | 14 | (31.1) | 11 | (32.4) |
| **Stage 3** | 26 | (28.9) | 9 | (20.0) | 11 | (32.4) |

Table 2. Trainees’ perception of the existing and expected formal academic mentoring program

|  | **Items** | **Existing** | **Expectation** | **P-value** |
| --- | --- | --- | --- | --- |
| 1 | The Academic supervisor selected by study  program | 5.90 (1.38) | 5.85 (1.37) | 0.619 |
| 2 | The Study program appoint academic supervisor  soon after trainee admission | 5.92 (1.36) | 5.88 (1.33) | 0.318 |
| 3 | Trainees submit the expected name of academic  supervisor to the study program | 3.81 (2.02) | 5.04 (1.80) | 0.000 |
| 4 | The Academic supervisor assess the adaptability of the  new trainees and help them in the adaptation process during study period | 5.01 (1.46) | 5.77 (1.23) | 0.000 |
| 5 | Substitution of the academic supervisors is  possible if both parties feels that the relationship between the two is not going well | 4.77 (1.60) | 5.61 (1.37) | 0.000 |
| 6 | The study program requires the academic  supervisors to meet with a trainee at least once  a semester | 5.32 (1.54) | 5.80 (1.32) | 0.000 |
| 7 | The academic supervisor conduct routine  evaluation of trainee’s academic performance | 5.11 (1.51) | 5.80 (1.26) | 0.000 |
| 8 | The academic supervisor help trainee identify  their strength and weakness | 5.07 (1.51) | 5.80 (1.33) | 0.000 |
| 9 | The academic supervisor provide information on  access to resources and support in career  planning | 5.19 (1.52) | 5.87 (1.30) | 0.000 |
| 10 | The academic supervisor provide information on  access to resources and counseling services | 5.21 (1.48) | 5.87 (1.35) | 0.000 |
| 11 | The academic supervisor know the trainee’s life  situation and its impact on education and  performance | 5.16 (1.48) | 5.78 (1.31) | 0.000 |
| 12 | The academic supervisor understand trainee’s  potential and interest | 5.08 (1.44) | 5.81 (1.27) | 0.000 |
| 13 | The academic supervisor easy to meet and  contacted when needed | 5.71 (1.23) | 6.02 (1.21) | 0.000 |
| 14 | The academic supervisor facilitate trainee to  become professional and competent | 5.59 (1.29) | 5.92 (1.23) | 0.000 |
| 15 | The academic supervisor help trainee to expand  their network and career experience | 5.40 (1.36) | 5.88 (1.26) | 0.000 |
| 16 | The academic supervisor help trainee to chose  research topic | 5.68 (1.35) | 5.80 (1.36) | 0.000 |
| 17 | Having an academic supervisor help trainee through  educational process | 5.19 (1.48) | 5.89 (1.26) | 0.000 |
| 18 | During the covid-19 pandemic, the academic  supervisor provided support and identified  problems such as stress and burnout in residents | 5.00 (1.53) | 5.80 (1.33) | 0.000 |
